# Blood Oxygenation Level Dependent Magnetic Resonance Imaging (MRI), Dynamic Contrast Enhanced MRI, and Diffusion Weighted MRI for Benign and Malignant Breast Cancer Discrimination: A Preliminary Experience

**DOI:** 10.3390/cancers13102421

**Published:** 2021-05-17

**Authors:** Roberta Fusco, Vincenza Granata, Mauro Mattace Raso, Paolo Vallone, Alessandro Pasquale De Rosa, Claudio Siani, Maurizio Di Bonito, Antonella Petrillo, Mario Sansone

**Affiliations:** 1 Radiology Division, Istituto Nazionale Tumori—IRCCS—Fondazione G. Pascale, 80131 Naples, Italy; r.fusco@istitutotumori.na.it (R.F.); m.mattaceraso@istitutotumori.na.it (M.M.R.); p.vallone@istitutotumori.na.it (P.V.); a.petrillo@istitutotumori.na.it (A.P.); 2 Department Electrical Engineering and Information Technologies, Universita’ Degli Studi DI Napoli Federico II, 80125 Naples, Italy; alessandro.derosa@studenti.unina.it (A.P.D.R.); msansone@unina.it (M.S.); 3 Senology Surgical Division, Istituto Nazionale Tumori—IRCCS—Fondazione G. Pascale, 80131 Naples, Italy; c.siani@istitutotumori.na.it; 4 Pathology Division, Istituto Nazionale Tumori—IRCCS—Fondazione G. Pascale, 80131 Naples, Italy; m.dibonito@istitutotumori.na.it

**Keywords:** breast cancer, hypoxia, perfusion, BOLD MRI, DCE-MRI

## Abstract

**Simple Summary:**

The aim of the study is to combine blood oxygenation level dependent magnetic resonance imaging (BOLD-MRI), dynamic contrast enhanced MRI (DCE-MRI), and diffusion weighted MRI (DW-MRI) in differentiation of benign and malignant breast lesions. The results suggest that the combined use of DCE-MRI, DW-MRI and/or BOLD-MRI does not provide a dramatic improvement compared to the use of DCE-MRI features alone, in the classification of breast lesions. However, an interesting result was the negative correlation between R_2_* and *D*.

**Abstract:**

Purpose. To combine blood oxygenation level dependent magnetic resonance imaging (BOLD-MRI), dynamic contrast enhanced MRI (DCE-MRI), and diffusion weighted MRI (DW-MRI) in differentiation of benign and malignant breast lesions. Methods. Thirty-seven breast lesions (11 benign and 21 malignant lesions) pathologically proven were included in this retrospective preliminary study. Pharmaco-kinetic parameters including *K*^trans^, *k*^ep^, *v*_e_, and *v*_p_ were extracted by DCE-MRI; BOLD parameters were estimated by basal signal S0 and the relaxation rate R_2_*; and diffusion and perfusion parameters were derived by DW-MRI (pseudo-diffusion coefficient (*D_p_*), perfusion fraction (*f*_p_), and tissue diffusivity (*D_t_*)). The correlation coefficient, Wilcoxon-Mann-Whitney U-test, and receiver operating characteristic (ROC) analysis were calculated and area under the ROC curve (AUC) was obtained. Moreover, pattern recognition approaches (linear discrimination analysis and decision tree) with balancing technique and leave one out cross validation approach were considered. Results. R_2_* and D had a significant negative correlation (−0.57). The mean value, standard deviation, Skewness and Kurtosis values of R_2_* did not show a statistical significance between benign and malignant lesions (*p* > 0.05) confirmed by the ‘poor’ diagnostic value of ROC analysis. For DW-MRI derived parameters, the univariate analysis, standard deviation of *D*, Skewness and Kurtosis values of D* had a significant result to discriminate benign and malignant lesions and the best result at the univariate analysis in the discrimination of benign and malignant lesions was obtained by the Skewness of D* with an AUC of 82.9% (*p*-value = 0.02). Significant results for the mean value of *K*^trans^, mean value, standard deviation value and Skewness of *k*_ep_, mean value, Skewness and Kurtosis of *v*_e_ were obtained and the best AUC among DCE-MRI extracted parameters was reached by the mean value of *k*_ep_ and was equal to 80.0%. The best diagnostic performance in the discrimination of benign and malignant lesions was obtained at the multivariate analysis considering the DCE-MRI parameters alone with an AUC = 0.91 when the balancing technique was considered. Conclusions. Our results suggest that the combined use of DCE-MRI, DW-MRI and/or BOLD-MRI does not provide a dramatic improvement compared to the use of DCE-MRI features alone, in the classification of breast lesions. However, an interesting result was the negative correlation between R_2_* and *D*.

## 1. Introduction

Cancer is the first or second leading cause of death in 112 of 183 countries and ranks third or fourth in a further 23 countries. Breast cancer in women is the most commonly diagnosed cancer and the leading cause of cancer death [1]. However, an early diagnosis of breast cancer reduces mortality.

Magnetic resonance imaging (MRI) allows quantifying biological, physiological, and pathological processes at the cellular and molecular level and provides information on key processes in cancer development and progression. Several researches have assessed the MRI role in the characterization of tumor properties such as vessel permeability, cellularity, and chemical composition [2,3,4,5,6]. Recently, one feature that is gaining increasing interest for tumor microenvironment characterization is hypoxia, a condition of low oxygenation, which is present in many solid tumors as rapidly proliferating cells outgrow the existing vasculature [7,8]. This propriety can be quantified in MRI following the differences between the magnetic susceptibility of oxyhemoglobin (diamagnetic) and deoxyhemoglobin (paramagnetic): This phenomenon is known as the blood oxygen level dependent (BOLD) effect [9,10,11,12]. Fractional oxygenation of blood changes are expected to affect *T*_2_* measurements in the vascular spaces and their neighborhoods. Literature results in breast cancer indicated an indirect association between *T*_2_* variations and tissue oxygenation [13,14].

Today, the standard acquisition protocol in breast cancer evaluation includes the dynamic contrast enhanced (DCE) MRI exam that requires a contrast agent intravenous injection allowing to highlight morphology, shape of breast lesions, and to depict areas with increased blood perfusion with intense contrast enhancement [15]. 

DCE-MRI provides information on morphology and vascularization of the tumor [16,17,18,19]. BOLD MRI gives information on blood hemoglobin oxygenation including the contribution of blood volume, hematocrit, flow, hemoglobin oxygen saturation, as well as pH, 2,3 DPG [20,21]. 

Moreover, diffusion weighted MRI (DW-MRI) sequences can be used in the MRI acquisition protocol for breast cancer assessment to depict water diffusion mobility, cellular density, and vessel structure [22,23,24]. Typically, breast cancer due to higher cellular density exhibits restricted water diffusion corresponding a low apparent diffusion coefficient (ADC) value by DWI-MRI [25,26,27,28]. ADC values have proven useful in the differentiation of breast cancer [25,26,27,28]. Moreover, the use of the intravoxel incoherent motion model (IVIM) in DWI-MRI data analysis provides information on cellularity, diffusion, and perfusion of tumors [29,30] using multi-b-value diffusion weighted images and bi-exponential curve fit [31]. Pseudo-diffusivity ( $D_{p}$ indicated also with D*), perfusion fraction ( $f_{p}$ ), and tissue diffusivity ( $D_{t}$ ) can be calculated by IVIM [29,30,31,32]. 

In this study, we combined DCE-MRI, BOLD-MRI, and DW-MRI features including model based parameters by DCE-MRI data and IVIM parameters by DW-MRI data, after automatic registration and preprocessing of three volumes, to assess the accuracy in differentiation of benign and malignant breast lesions. 

## 2. Methods

### 2.1. Patient Characteristics

National Cancer Institute of Naples Local Ethical Committee approved the study with the deliberation n. 617 of 9 August 2016. Therefore, each patient signed the informed consent. The study was performed in accordance with an up-to-date Declaration of Helsinki version and International Conference on Harmonization of Good Clinical Practice Guidelines. 

We included in the analysis patients that performed a breast MRI exam including DCE-MRI, DW-MRI, and BOLD-MRI sequences to suspected breast lesions characterization. In this retrospective study, in a 1-year period from January–December 2020, we included 37 women with 11 benign and 21 malignant lesions, their age ranging from 30 to 77 years (median, 53; standard deviation 11). 

Exclusion criteria were: Patients undergoing chemotherapy and radiotherapy; patients without histopathological tests. 

### 2.2. MRI Protocol

The MR 1.5 T scanner (Magnetom Symphony, Siemens Medical System, Erlangen, Germany) equipped with a 16-element breast dedicated coil was used to acquire the MRI examinations.

Ten series including one before and nine series after intravenous injection of 0.1 mmol/kg body weight of a positive paramagnetic contrast material (Gd-DOTA; Dotarem, Guerbet, Roissy CdG Cedex, France) were acquired. The temporal interval between two successive scans was 56 s. An automatic injection system was used (Spectris Solaris EP MR, MEDRAD, Inc., Indianola, PA, USA) and the injection flow rate was set to 2 mL/s, followed by a flush of 10 mL saline solution at the same rate. 

DW-MRI included 7 fat suppressed scans in axial plane with different *b*-values (0, 50, 100, 150, 400, 800, and 1000 s/mm^2^), acquired with spectral adiabatic inversion recovery (SPAIR).

The BOLD-MRI included 10 fat suppressed scans in sagittal plane with different TE values (4, 8, 12, 16, 20, 24, 28, 32, 36, and 40 ms) acquired with SPAIR at breath hold (BH).

Details of the MRI sequences were provided in Table 1.

### 2.3. Manual ROI and Volume Coregistration

Two expert radiologists with 15 and 20 years of breast imaging experience, in consensus, manually drew slice by slice regions of interest (ROIs) following the margins of breast lesion, in order to obtain the volume of interest. In DCE-MRI, the ROIs were drawn on a third series considering the arterial phase of contrast agent uptake. In BOLD-MRI, the ROIs were defined on the R_2_* image (BOLD) at TE of 16 ms. In DW-MRI, the ROIs were defined on diffusion weighted images at the highest *b*-value (1000 s/mm^2^). Then, the validation of the lesion contours was made by another expert radiologist with 30 years of breast imaging experience.

The volume intersection and a 3D linear interpolation was performed in order to align DCE-MRI, DW-MRI, and BOLD-MRI volumes on a common grid. For the subsequent analysis, only voxels included in all datasets were considered. The post-processing was performed using MATLAB (The MathWorks, Inc., Natik, MA, USA).

### 2.4. BOLD Image Analysis

Per each voxel of volume of interest and considering all echoes, two features were extracted from BOLD-MRI data assuming that the $T_{2}^{*}$ mono-exponential decay of the signal follows this equation [10,11,12,13,14]:

(1) $$S\left(TE\right)=S_{0}e^{-TE/T_{2}^{*}}$$

where S(TE) is the signal intensity at a given echo time, $S_{0}$ is the signal intensity at TE=0 and represents water proton density. The extracted BOLD-MRI parameters are: $S_{0}$ and $R_{2}^{*}$ ( $1/T_{2}^{*}$ ), the relaxation rate. 

Indeed Equation (1) can be written as follows:

(2) $$S\left(TE\right)=S_{0}e^{-R_{2}^{*}TE}$$

The echo images were used as input for fitting and calculation of $S_{0}$ and $R_{2}^{*}$ values using the conventional non-linear least squares (NLLS) [33] algorithm. 

### 2.5. DW-MRI Image Analysis 

Per each voxel of volume of interest, three features (pseudo-diffusivity ( $D_{p}$ indicated also with D*, perfusion fraction (*f*), and tissue diffusivity ( D )) were extracted by DW-MRI data using the IVIM model and all b-values. A bi-exponential model and the conventional NLLS [28,29,30,31,32,33] was used to estimate the parameters with the following equation:

(3) $$\frac{S_{b}}{S_{0}}=f_{p\cdot }\exp\left(-b\;\cdot D_{p}\right)+\left(1-f\right)\cdot \exp\left(-b\cdot D\right)$$

### 2.6. DCE-MRI Image Analysis 

Per each voxel of volume of interest, three quantitative model based features were extracted from DCE-MRI. The contrast medium concentration in time is typically modelled using the extended Tofts model [34,35,36].

(4) $$C_{t}\left(t,\;K^{\mathrm{trans}},\;k_{\mathrm{ep}}\right)=C_{p}\left(t\right)*K^{\mathrm{trans}}.{\;e}^{-k_{\mathrm{ep}}t}+v_{p}.\;C_{p}\left(t\right)$$

where 𝐶_t_ (𝑡) is the concentration of contrast medium in the tissue; 𝐶_𝑝_(𝑡) is the concentration of contrast medium within the plasma; 𝐾^trans^ is the volume transfer constant (the diffusion rate constant from EES to plasma); *v*_𝑝_ is the volume fraction occupied by plasma. We assumed the bi-exponential arterial input function proposed by Weinmann et al. [37]:

(5) $$C_{p}\left(t\right)=d\;\left(a_{1}\exp(-m_{1}t\right)+a_{2}\;\exp\left(-m_{2}t\right))$$

where 𝑑 is the administered dose (mL/kg), 𝑎_1_ = 3.99 kg/L, 𝑎_2_ = 4.78 kg/L, 𝑚_1_ = 0.144 min^−1^, and 𝑚_2_ = 0.0111 min^−1^. The contrast medium concentration was calculated using the time intensity curve by Schabel et al. [38] with a fixed pre-contrast longitudinal relaxation time, 𝑇_1,0_ of 820ms, appropriate for breast parenchyma.

### 2.7. Reference Standard and Pathological Methods

The reference standard was the pathology from a surgical specimen for malignant lesions and pathology from a surgical specimen or core needle biopsy for benign lesions. Breast tumors were classified according to the American Joint Committee on Cancer staging. Malignant lesions included the ductal carcinoma in situ, invasive cancers tumors. Benign lesions included lobular carcinoma in situ, fibroadenoma, ductal hyperplasia, dysplasia, cysts, fibrosis, and phyllodes tumor.

### 2.8. Statistical Analysis

The analysis of the extracted parameters was made as both voxel based and ROI based. The mean, standard deviation, Skewness and Kurtosis values were calculated as representative values of the extracted parameters.

#### 2.8.1. Univariate Analysis

The Mann-Whitney U-test was used to assess the differences in DCE-MRI, DW-MRI, and BOLD-MRI derived parameters to differentiate benign and malignant lesions. The diagnostic performance of extracted parameters was assessed using the receiver operating characteristic (ROC) curve analysis. The best cut-off, area under the curve (AUC), sensitivity, specificity, positive predictive value (PPV), and negative predictive value (NPV) were calculated. 

A *p*-value < 0.05 was considered as significant for univariate analysis. Statistical analysis was performed with the Rstudio software [39]. 

#### 2.8.2. Multivariate Analysis

At the multivariate analysis, pattern recognition approaches (linear discriminant analysis (LDA) and decision tree (DT)) were considered.

The leave-one-out validation approach was used as a cross-validated approach and median values of AUC, accuracy, sensitivity, and specificity were reported.

To help balance the two classes (benign and malignant lesions), the adaptive synthetic sampling (ADASYN) approach was used. The adaptive synthetic sampling (ADASYN) approach is one of the most successful advanced over-sampling approaches. It is an extension of the synthetic minority over-sampling technique (SMOTE) [40,41], which [42] tackles the class imbalance problem by creating linear interpolations between randomly selected minority class samples and their neighbors of the same class. The essential idea of ADASYN is to prioritize samples near decision boundaries and to focus on these hard-to-learn minority class samples by assigning weights calculated per sample, according to their level of difficulty in learning, as the ratio of neighbors belonging to the majority class [43].

A *p*-value < 0.05 was considered as significant for the univariate analysis. The statistical analysis was performed with the Rstudio software [39]. 

## 3. Results

### 3.1. Univariate Analysis Results

Table 2 shows the mean and standard deviation value of extracted parameters voxel by voxel.

Table 3 shows the mean and standard deviation value of extracted parameters ROI based.

Table 4 shows the *p*-value at the Wilcoxon-Mann-Whitney U-test for each extracted parameter in the voxel by voxel analysis. The significant results in the discrimination of benign and malignant lesions were obtained by Skewness of *S*_0_, mean value of *K^trans^*, mean value, standard deviation value and Skewness of *k*_ep_, mean value, Skewness and Kurtosis values of *v*_e_, standard deviation value of *D*, Skewness and Kurtosis values of D*. The ROI based analysis had similar results. 

**Figure 1:**
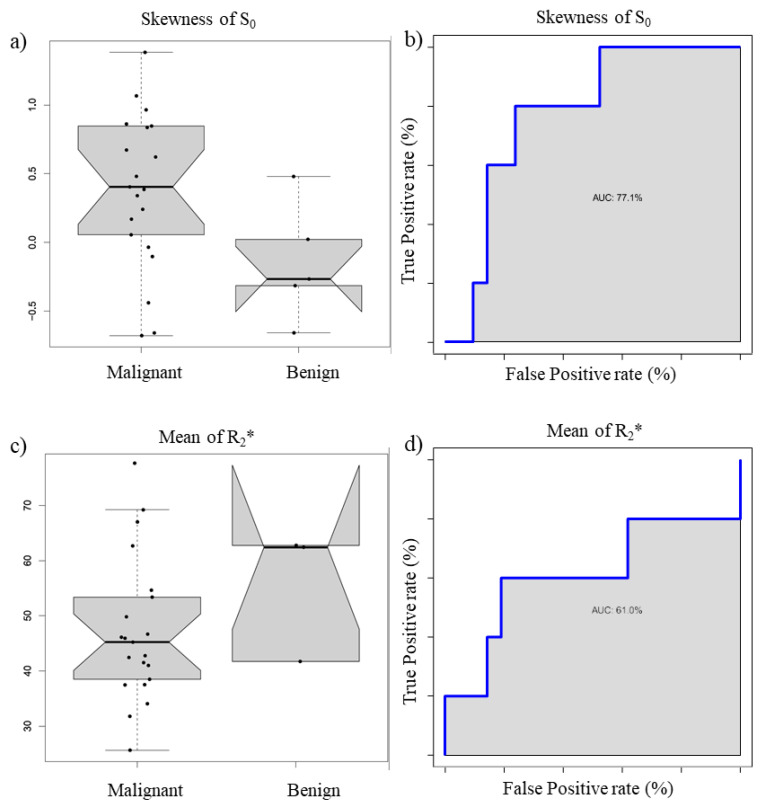
Boxplot and ROC curve of S_0_ Skewness (**a**,**b**) and R_2_* mean value (**c**,**d**).

Figure 1 reports the boxplot and ROC curve of *S*_0_ Skewness (a and b) and R_2_* mean value (c and d). 

**Figure 2:**
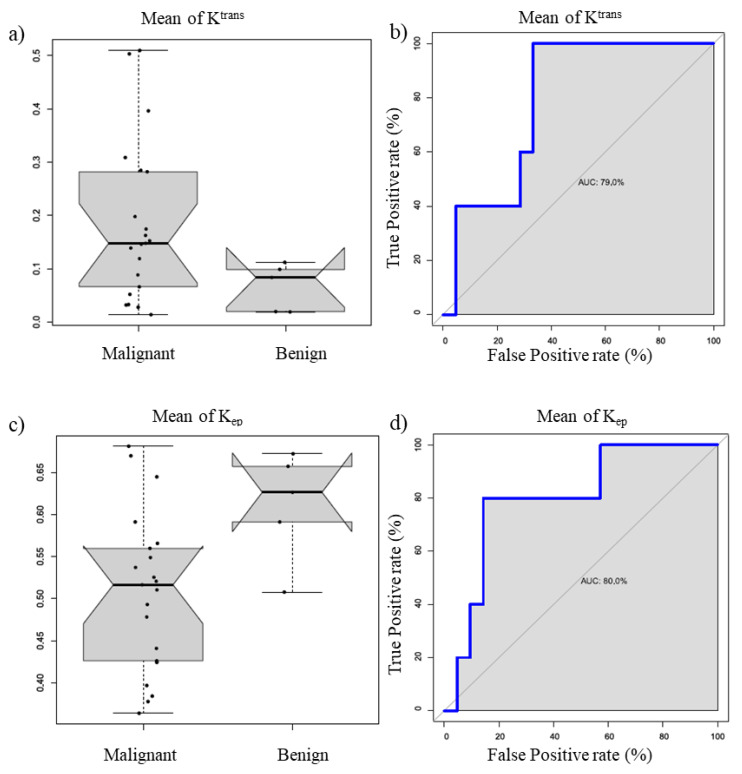
Boxplot and ROC curve of K^trans^ mean value (**a**,**b**) and k_ep_ mean value (**c**,**d**).

Figure 2 reports the boxplot and ROC curve of *K*^trans^ mean value (a and b) and k_ep_ mean value (c and d). 

**Figure 3:**
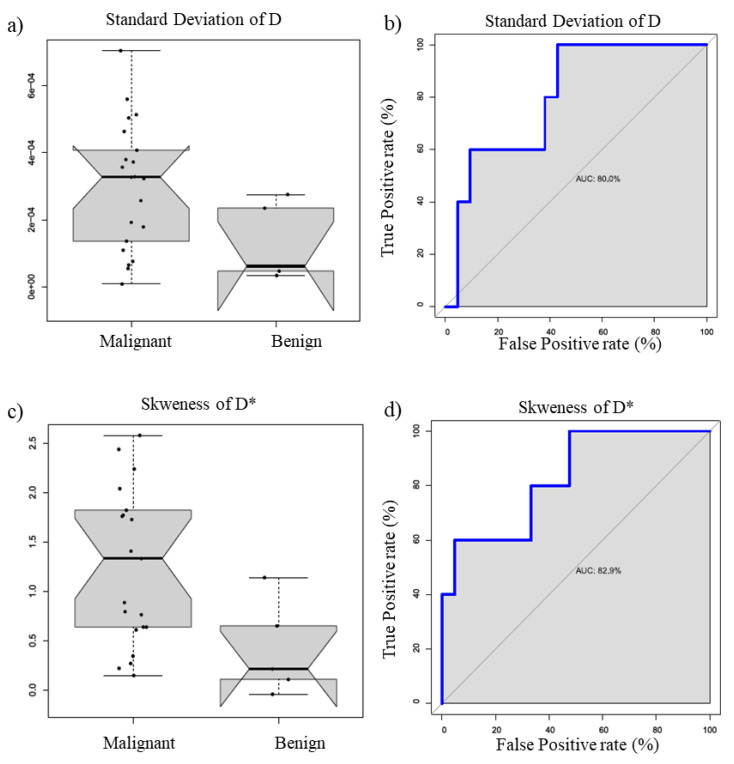
Boxplot and ROC curve of K^trans^ mean value (**a**,**b**) and k_ep_ mean value (**c**,**d**).

Figure 3 reports the boxplot and ROC curve of D standard deviation value (a and b) and D* Skewness D* (c and d). 

The best result at the univariate analysis in the discrimination of benign and malignant lesions was obtained by the Skewness of D* with an AUC of 82.9% (*p*-value = 0.02). 

**Figure 4:**
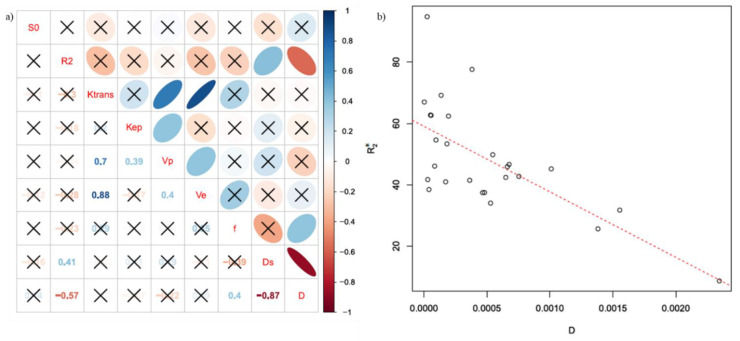
(**a**) Representative diagram of correlation coefficients between BOLD, DCE, and DWI extracted parametersat the voxel by voxel analysis; in (**b**) the scatter plot for the couple of parameters with the best correlation (R_2_* and *D*).

Figure 4 reports in (a) the representative diagram of correlation coefficients between BOLD, DCE, and DWI extracted parameters at the voxel by voxel analysis, while in (b) the scatter plot for the couple of parameters with the best correlation. 

**Figure 5:**
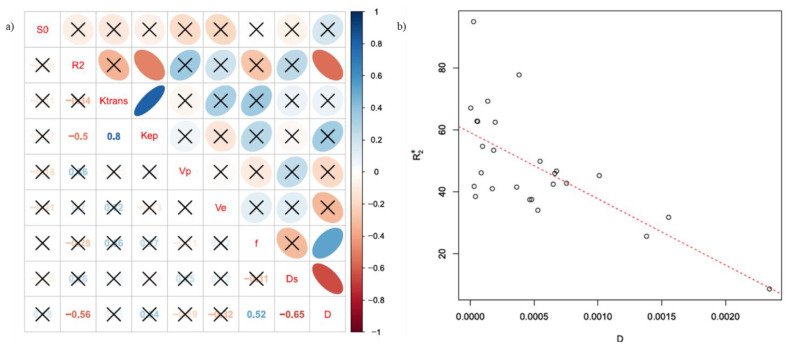
(**a**) Representative diagram of correlation coefficients between BOLD, DCE, and DWI extracted parameters at the ROI based analysis; in (**b**) the scatter plot for the couple of parameters with the best correlation (R_2_* and *D*).

Figure 5 reports in (a) the representative diagram of correlation coefficients between BOLD, DCE, and DWI extracted parameters at the ROI based analysis, while in (b) the scatter plot for the couple of parameters with the best correlation. 

R_2_* and D showed the best significant correlation coefficient equal to −0.57 at the voxel by voxel analysis and −0.56 at the ROI based analysis. 

### 3.2. Multivariate Analysis Results

**Figure 6:**
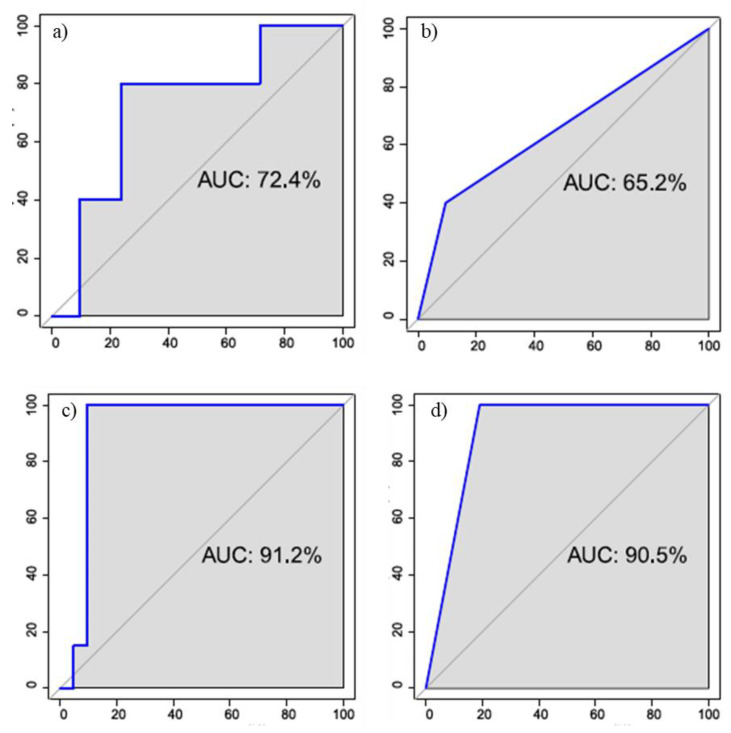
ROC curve for the best classifier to discriminate benign and malignant lesions for the voxel based (**a**,**c**) and ROI based analysis (**b**,**d**)

In the case of voxel by voxel, the best performance was obtained considering only the parameters DCE and using an LDA classifier (accuracy = 0.88, sensitivity = 0.90, specificity = 0.80, AUC = 0.72) (Figure 6a)). In the case of ROI based analysis, the best result is obtained with a decision tree and with a combination of BOLD parameters and DWI (accuracy = 0.81, sensitivity = 0.90, specificity = 0.40, AUC = 0.65) (Figure 6b)). 

With the application of the SMOTE algorithm the number of subjects with benign lesions has been artificially increased, thus obtaining a balance of classes. Moreover, in this case, in the voxel by voxel analysis the best results was obtained on the DCE parameters using the LDA classifier (accuracy = 0.95, sensitivity = 0.90, specificity = 1.00, AUC = 0.91) (Figure 6c)). For the ROI based analysis, the best performance was achieved with the decision tree and always considering the DCE parameters (accuracy = 0.90, sensitivity = 0.81, specificity = 1.00, AUC = 0.91) (Figure 6d)). 

## 4. Discussion

According to our knowledge, no previous study has combined DCE-MRI, BOLD-MRI, and DW-MRI parameters including model based parameters evaluated by DCE-MRI data and IVIM parameters by DW-MRI data, after automatic registration and preprocessing of three volumes, in order to evaluate the accuracy in the differentiation of benign and malignant breast lesions. In this study, we applied the combination of DCE-MRI, BOLD-MRI, and DW-MRI techniques on a population of 37 patients with confirmed breast cancer.

Among the BOLD parameters, the parameter that gives more pathological information is R_2_*, which increases with the deoxyhemoglobin concentration identifying a more hypoxic area (typical of malignant lesion) [44]. In this study, the mean value, standard deviation, Skewness and Kurtosis values of R_2_* did not show a statistically significant difference between benign and malignant lesions confirmed by the ‘poor’ AUC values, probably linked to breast cancer heterogeneity, movement artefacts, limited number of patients. Some studies have shown a lack of utility of R_2_* alone, while the delta of R_2_* has shown promise in both clinical and pre-clinical investigations [37,45,46]. Our results suggested that the univariate analysis of BOLD-MRI derived parameter did not allow the discrimination of benign and malignant breast lesions. This result was in accordance with our previous study [47] that reported no significant finding in the discrimination of benign and malignant breast lesions considering BOLD parameters alone.

For DW-MRI derived parameters, the univariate analysis, standard deviation of *D*, Skewness and Kurtosis values of D* had significant results to discriminate benign and malignant lesions. The best result was obtained by the Skewness of D* with an AUC of 82.9% (*p*-value = 0.02). Our results are in accordance with findings of Liu et al. [43] that have reported that IVIM quantitative parameters are helpful to discriminate benign and malignant breast lesions. Mao et al. [42] reported that IVIM parameters could help improve the specificity and accuracy to identify malignant lesions. The D-value is most relevant and valuable in predicting the grading of malignant breast lesions.

Several authors have combined DCE and DW-MRI data in breast cancer to different aims. Rahbar et al. [48] developed a model including DCE and DW-MRI features to differentiate a high nuclear grade (HN) from non-HNG ductal carcinoma in situ (DCIS) in vivo: DCE and DW-MRI imaging features could identify patients with high risk DCIS. Partridge et al. [49] showed that ADC could improve the PPV of breast MRI for lesions of varied types and sizes. Jena et al. [50] have tried to evaluate the combined effect of capillary permeability (𝐾^trans^) and tissue cellularity (ADC) on the diagnostic accuracy for differentiating benign and malignant breast lesions by incorporating these parameters in a routine clinical protocol for breast MRI. Fusco et al. [5] reported that the combination of DWI and DCE-MRI did not increase the sensitivity and specificity in the classification of breast lesions. DCE-MRI alone gave the same performance as in the combination with DW-MRI.

We obtained significant results for the mean value of *K*^trans^, mean value, standard deviation value and Skewness of *k*_ep_, mean value, Skewness and Kurtosis values of *v*_e_. The best AUC among DCE-MRI extracted parameters was reached by the mean value of *k*_ep_ and was equal to 80.0%.

However, in this manuscript, the best diagnostic performance in the discrimination of benign and malignant lesions was obtained at the multivariate analysis considering the DCE-MRI parameters alone with an AUC = 0.91 when the balancing technique was considered. The integration of DCE-MRI, DW-MRI, and BOLD-MRI did not improve the diagnostic performance. 

Our findings showed that R_2_* and D had a significant negative correlation. This finding in accordance with Lee at al. [51] indicated that rapid R_2_* relaxation rates are associated with lower diffusion rates, which is consistent with the action of macromolecules that cause signal dephasing (through residual dipolar coupling) and also inhibit the free motion of water molecules.

## 5. Conclusions

The current study had several limitations: Data were derived from only one oncological center, a small group of women that may influence the generalization of the results. However, we considered this study as a preliminary report with the objective to integrate DCE-MRI, DW-MRI, and BOLD-MRI in breast lesion classification, which is the retrospective nature of the study. In this study, the technique to distinguish the subtypes of breast lesions is not analyzed but this could be a future endpoint.

Although preliminary, our results seem to suggest that the combined use of DCE-MRI, DW-MRI, and/or BOLD-MRI did not provide a dramatic improvement compared to the use of DCE-MRI features alone, in the classification of breast lesions. Another interesting result was the negative correlation between R_2_* and *D*. 

## Figures and Tables

**Table 1:** MRI sequence parameters.

Settings	DCE-MRI	BOLD-MRI	DW-MRI	Units
TR/TE/FA	5.08/2.39/15	358/4–40.187/40	7700/128/90	ms/ms/deg
Plane	Axial	Sagittal	Axial	
FOV	500 × 500	211 × 260	203 × 400	mm^2^
Matrix size	384 × 384	104 × 128	120 × 236	pixel
Pixel spacing	0.885 × 0.885	2.03 × 2.03	1.69 × 1.69	mm^2^
Slice thickness	1.60	6	4	mm
Gap between slices	0	7.2	6	mm
No. of slices	128	80	168	-

**Table 2:** Mean and standard deviation value of extracted parameters voxel by voxel.

		*S* _0_[A.U.]H	R_2_* [Hz]	*D* [10^4^ mm^2^ s^−1^]	*f* [%]	D* [10^4^ mm^2^ s^−1^]	*K*^trans^ [1/min]	*k*_ep_ [1/min]	*v*_p_ [%]	*v*_e_ [%]
Benign	Mean	506.0	54.1	5.3	9.1	48.6	0.1	0.8	0.2	12.8
	Standard Deviation	57.3	31.7	10.2	4.9	15.2	0.0	0.3	0.2	5.3
Malignant	Mean	578.0	45.2	4.9	9.4	34.6	0.2	0.5	0.5	36.4
	Standard Deviation	242.0	13.0	4.3	3.8	14.1	0.1	0.2	0.4	15.5
Total	Mean	564.0	48.5	5.0	9.3	37.3	0.2	0.6	0.4	31.9
	Standard Deviation	220.0	17.5	5.6	3.9	15.1	0.1	0.3	0.4	16.9

**Table 3:** Mean and standard deviation value of extracted parameters ROI based.

		*S*_0_ [A.U.]	R_2_* [Hz]	*D* [10^4^ mm^2^ s^−1^]	f [%]	D* [10^4^ mm^2^ s^−1^]	*K*^trans^ [1/min]	*k*_ep_ [1/min]	*v*_p_ [%]	*v*_e_ [%]
Benign	Mean	487.0	47.8	5.1	8.6	34.8	0.0	0.1	0.3	46.2
	Standard Deviation	62.9	26.0	10.2	5.3	24.8	0.0	0.1	0.3	30.0
Malignant	Mean	555.0	41.9	4.3	9.3	24.4	0.2	0.3	0.2	44.4
	Standard Deviation	233.0	11.3	3.9	5.1	23.4	0.2	0.2	0.3	19.2
Total	Mean	542.0	43.0	4.4	9.2	26.4	0.1	0.3	0.2	44.7
	Standard Deviation	212.0	14.7	5.4	5.0	23.6	0.1	0.2	0.3	20.6

**Table 4:** *p*-value at the Wilcoxon-Mann-Whitney U-test (bolding indicates significance *p* ≤ 0.05).

*p* Value at Wilcoxon-Mann-Whitney U-Test	Mean	Standard Deviation	Skewness	Kurtosis
S_0_	0.90	0.71	0.05	0.11
R_2_∗	0.08	0.75	0.14	0.09
K^trans^	**0.05**	0.22	0.37	0.20
k_ep_	**0.04**	**0.04**	**0.03**	0.18
v_p_	0.34	0.18	0.41	0.37
v_e_	**0.00**	0.07	**0.01**	**0.01**
f	0.95	0.22	0.06	0.22
D	0.22	**0.04**	0.31	0.06
D^∗^	0.08	0.53	**0.02**	**0.04**

## Data Availability

All data are reported in the manuscript.
